# UHPLC-MS Chemical Fingerprinting and Antioxidant, Enzyme Inhibition, Anti-Inflammatory In Silico and Cytoprotective Activities of *Cladonia chlorophaea* and *C. gracilis* (Cladoniaceae) from Antarctica

**DOI:** 10.3390/antiox12010010

**Published:** 2022-12-21

**Authors:** Alfredo Torres-Benítez, José Erick Ortega-Valencia, Marta Sánchez, Mathias Hillmann-Eggers, María Pilar Gómez-Serranillos, Gabriel Vargas-Arana, Mario J. Simirgiotis

**Affiliations:** 1 Instituto de Farmacia, Facultad de Ciencias, Universidad Austral de Chile, Campus Isla Teja, Valdivia 5090000, Chile; 2 Tecnológico Nacional de México, Instituto Tecnológico de Tlalnepantla, Av. Instituto Tecnológico, S/N. Col. La Comunidad, Tlalnepantla de Baz 54070, Mexico; 3 Departamento de Farmacología, Farmacognosia y Botánica, Facultad de Farmacia, Universidad Complutense de Madrid, Plaza Ramón y Cajal s/n, Ciudad Universitaria, 28040 Madrid, Spain; 4 Laboratorio de Química de Productos Naturales, Instituto de Investigaciones de la Amazonía Peruana, Avenue Abelardo Quiñones, Iquitos 16001, Peru; 5 Facultad de Industrias Alimentarias, Universidad Nacional de la Amazonía Peruana, Iquitos 16001, Peru

**Keywords:** *Cladonia*, secondary metabolites, antioxidant, enzyme inhibition, anti-inflammatory, cytoprotective, Antarctic lichens

## Abstract

The lichen species *Cladonia chlorophaea* and *C. gracilis* (Cladoniaceae) are widely distributed in the island archipelago of maritime Antarctica and represent a natural resource of scientific interest. In this work, the metabolomic characterization of the ethanolic extracts of these species and the determination of the antioxidant activity, enzymatic inhibition and anti-inflammatory potential of selected compounds on the 5-lipoxygenase enzyme by molecular docking and cytoprotective activity in the SH-SY5Y cell line were carried out. Nineteen compounds were identified by liquid chromatography coupled with quadrupole-time-of-flight mass spectrometry (UHPLC-ESI-QTOF-MS) in each of the species. The contents of phenolic compounds, antioxidant activity, the inhibition of cholinesterases (acetylcholinesterase and butyrylcholinesterase) and digestive enzymes (α-glucosidase and pancreatic lipase) were variable among species, with better results in *C. chlorophaea*. Molecular docking evidenced significant binding affinities of some compounds for the 5-lipoxygenase enzyme, together with outstanding pharmacokinetic properties. Both extracts were shown to promote cell viability and a reduction in reactive oxygen species production in an H_2_O_2_-induced oxidative stress model. This study contributes to the chemical knowledge of the *Cladonia* species and demonstrates the biological potential for the prevention and promising treatment of central nervous system pathologies, inflammatory disorders and metabolic alterations.

## 1. Introduction

**Figure 1:**
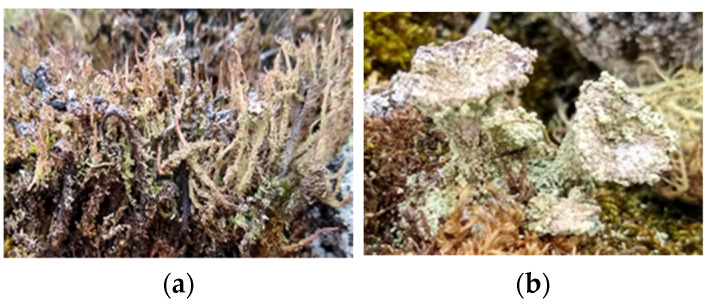
U Lichens thallus in the polar tundra of King George Island, Maritime Antarctic: (**a**) *C. gracilis*; (**b**) *C. chlorophaea*.

Lichens are defined as a mutualistic relationship between a heterotrophic mycobiont and an autotrophic photobiont (algae and/or cyanobacteria) associated with an abundant and structured microbiome [1,2]. Antarctic lichens have aroused deep interest for their abundance, their sensitivity to environmental variables [3], their natural products and the abundance of particular bioactive compounds derived from the mycobiont that are organized in different chemical classes, such as depsides, depsidones, dibenzofurans, xanthones, terpenic derivatives, anthraquinones, etc., and synthesized through the acetyl-polymalonate (APP), shikimic acid (SAP) and mevalonic acid (MAP) pathways in the fungal component with multiple biological activities [4]. Species of the genus *Cladonia* (Clado- niaceae, Lecanorales) are characterized by an erect thallus consisting of podecia; primary thallus, persistent or absent; apothecia, absent or present and red or brown; and biatorine. They are usually found growing on mosses [5] (Figure 1).

Lichen secondary metabolites are widely studied by robust techniques such as ultra-high-resolution chromatography (UHPLC) coupled with a mass spectrometer, and more than 1000 bioactive compounds are currently registered [6]. As for the genus *Cladonia*, there is evidence of the potential of extracts with major compounds, such as atranorin and oxalic, propionic, tartaric, butyric, malonic, lactic, citric, maleic, fumaric, stytic, succinic, norstytic, usnic, lecanoric, orsellinic and furmarprotocetraric acids, that show significant antioxidant activity, as in the case of the species *Cladonia foliacea, C. fimbriata, C. digitata, C. verticillaris, C. furcata, C. pyxidata, C. rangiferina, C. uncialis, C. rappi, C. pocillum* and *C. clathrate* [7]; likewise, recent studies show the positive effect of extracts of Antarctic lichens for the enzymatic inhibition of cholinesterases (acetylcholinesterase and butyrylcholineste- rase) that influence the interruption of nerve cell communication [8]. As a complement, this work shows the effect of these natural products on the inhibition of other enzymes involved in metabolic alterations associated with the development of chronic non-communicable diseases.

The study of the effect of extracts has also been enhanced on precursor targets of alterations of inflammatory origin; one of them, arachidonate 5-lipoxygenase (5-LOX), is a soluble monomeric enzyme that contains 673 amino acids and has two domains in its structure. The smaller domain is the N-terminal domain, while the larger domain is the area comprising the catalytic C-terminus with a non-heme iron atom. 5-LOX catalyzes the formation of 5(S)-hydroperoxyeicosatetraenoic acid (5-HPETE) from arachidonic acid (AA) by incorporating molecular oxygen, thus generating the dehydration of 5-HPETE to leukotriene A4 (LTA4); the conversion of this leukotriene (LTA4) causes the synthesis of other leukotrienes that, together with LTA4, are important mediators in the inflammatory response [9]. Therefore, 5-LOX is considered one of the important enzymes in the inflammatory response, it being the main cause of pathologies such as asthma and other inflammatory disorders, such as allergic rhinitis and rheumatoid arthritis [9,10]. On the other hand, recent studies with extracts of lichens such as *Dactylina arctica, Nephromopsis stracheyi, Tuckermannopsis americana* and *Vulpicida pinastri* and compounds such as evernic acid confirm their potential as neuroprotective agents in cellular models of oxidative stress by H_2_O_2_ and murine models of Parkinson’s disease induced by MPTP (1-methyl-4-phenyl-1,2,3,6-tetrahydropyridine) [11,12].

In this work, we report the identification of the compounds present in the ethanolic extract of *C. chlorophaea* and *C. gracilis* species, their content of phenolic compounds, an- tioxidant activity and enzymatic inhibition on cholinesterases, α-glucosidase and pancreatic lipase, an in silico analysis of the anti-inflammatory potential on the 5-LOX enzyme and their cytoprotection activity against H_2_O_2_-induced oxidative stress.

## 2. Materials and Methods

### 2.1. Chemicals

Ultrapure water (˂5 µg/L TOC) obtained by a water purification system (Mili-Q Merck Millipore, Chile) was used. For mass spectrometry analysis, HPLC-grade methanol and MS-grade formic acid were used, obtained from J.T. Baker (Phillipsburg, NJ, USA). Gallic acid, Folin Ciocalteu commercial reagent, 2,4,6-tris(2-pyridyl)-s-triazine, sodium carbonate, acetic acid, ferric chloride hexahydrate, sodium acetate, Trolox, hydrochloric acid, 2,2′-Azobis(2-amidinopropane) dihydrochloride, absolute ethanol, fluorescein solution, phosphate buffer, acetylcholinesterase enzyme (AChE), butyrylcholinesterase enzyme (BChE), galantamine, Tris-HCl buffer, Ellman’s reagent (DTNB), acetylcholine, butyrylcholine, magnesium chloride, sodium chloride, alpha-glucosidase, 4-nitrophenyl α-d-glucopyranoside, acarbose, orlistat, pancreatic lipase, 4-nitrophenyl-dodecanoate, dimethyl sulfoxide (DMSO) and HPLC standard with a purity greater than 95% (usnic acid and atranorin) were obtained from Sigma (Sigma, St. Louis, MO, USA).

### 2.2. Lichen Material

The specimens of the lichens *Cladonia gracilis* (L.) Willd. (100 g) and *C. chlorophaea* Flörke ex Sommerf. (Cladoniaceae, Lecanorales) (100 g) were collected by A.T.-B. and M.J.S. on Ardley Island, King George Island, South Shetland Archipelago in February 2021. The specimens were determined by botanist Alfredo Torres-Benítez. The specimen numbers HL-01122021 (*C. gracilis*) and HL-01132021 (*C. chlorophaea*) were deposited at the Natural Products Laboratory of the Universidad Austral de Chile, Valdivia, Chile.

### 2.3. Preparation of Ethanolic Extracts

A total of 5 g of each lichen species was macerated with ethanol (three times with 30 mL each) via ultrasound at room temperature. Each extract was filtered, and the solutions were concentrated under reduced pressure at 38 °C to obtain two final gummy extracts.

### 2.4. LC Parameters and MS Parameters

The separation and identification of the compounds present in the lichen extracts were performed on a UHPLC-ESI-QTOF-MS system equipped with UHPLC Ultimate 3000 RS with Chromeleon 6.8 software (Dionex GmbH, Idstein, Germany) and Bruker maXis ESI-QTOF-MS with the software Data Analysis 4.0 (all Bruker Daltonik GmbH, Bremen, Germany). A total of 5 mg of each extract was dissolved in 2 mL of methanol for analysis and filtered with a polytetrafluoroethylene (PTFE) filter, and 10 µL was injected into the equipment. The chromatographic equipment consisted of a quaternary pump, an autosampler, a thermostated column compartment and a photodiode array detector. Elution was performed with a binary gradient system with eluent (A) 0.1% formic acid in the water, eluent (B) 0.1% formic acid in the acetonitrile and the gradient: 1% B isocratic (0–2 min), 1–5% B (2–3 min), 5% B isocratic (3–5 min), 5–10% B (5–8 min), 10–30% B (8–30 min), 30–95% B (319–38 min) and 1% B isocratic (39–50 min). Separation was carried out with a Thermo 5 μm C18 80 Å column (150 mm × 4.6 mm) at a flow rate of 1.0 mL/min. ESI-QTOF-MS experiments were recorded in negative ion mode, and the scan range was between 100 and 1200 *m*/*z*. Electrospray ionization (ESI) conditions included a capillary temperature of 200 °C, a capillary voltage of 2.0 kV, a dry gas flow rate of 8 L/min and a nebulizer pressure of 2 bar. The experiments were performed in automatic MS/MS mode. The structural characterization of secondary metabolites was based on HR full MS, fragmentation patterns and comparisons with the literature data.

### 2.5. Total Phenolic (TP) Content

The total phenolic content (TPC) of the extracts was measured by the Folin–Ciocalteu (FC) method [13,14]. Briefly, the test mixture was FC reagent (10%), the extract sample, Na_2_CO_3_ (7%), was kept for 90 min in darkness and absorbance was measured at 750 nm in a microplate reader (BioTek Instrument, Inc., Winooski, VT, USA). The results were expressed as mg of gallic acid per gram of dried lichen.

### 2.6. Antioxidant Activity

#### 2.6.1. Ferric-Reducing Antioxidant Power (FRAP) Assay

The FRAP assay was performed following a method previously described in Karadag et al. and Parra et al. [15,16]. Briefly, the assay mixture was FRAP reagent, the extract sample was kept for 30 min in darkness and absorbance was measured at 593 nm in a microplate reader (BioTek Instrument, Inc., Winooski, VT, USA). Trolox was used as the reference compound. The results were expressed as micromoles of Trolox equivalents per gram of dried lichen.

#### 2.6.2. Oxygen Radical Absorbance Capacity (ORAC) Assay

The assay was performed following a method previously described by Huang et al. [17]. Briefly, the assay mixture was fluorescein, Trolox and phosphate buffer, the extract sample was incubated for 30 min at 37 °C and AAPH solution was added as the peroxyl generator. Fluorescence intensity was measured at 520 nm on a microplate reader (BioTek Instrument, Inc., Winooski, VT, USA) every 2 min for 1 h and 30 min at an excitation/emission wavelength of 485/520 nm. Trolox was used as the reference compound. The results were expressed as micromoles of Trolox equivalents per gram of dried lichen.

#### 2.6.3. DPPH Scavenging Activity

The assay was determined as previously reported [18]. Briefly, the assay mixture was a solution of DPPH (400 mM), the standard or extract sample, as appropriate, was kept for 30 min in the dark and absorbance was measured at 515 nm on a microplate reader (BioTek Instrument, Inc., Winooski, VT, USA). Gallic acid was used as a reference standard. The results were expressed as IC_50_ values (μg of lichen/mL).

### 2.7. Enzymatic Inhibitory Activity

#### 2.7.1. Cholinesterase Inhibition

According to Ellman’s method [19], the inhibition activity was determined. DTNB solution (3mM), AChE (0.26 U/mL) and BChE (0.26 U/mL) enzyme solution were added, as appropriate, Tris-HCl buffer (50 mM, pH 8.0) and solutions of the extracts at different concentrations were incubated for 20 min at 25 °C, the substrates acetyl thiocholine iodide (15 mM) and butyryl thiocholine chloride (15 mM) were added, as appropriate, and absorbance was measured at 412 nm on a microplate reader (BioTek Instrument, Inc., Winooski, VT, USA). The results were expressed as IC_50_ values (μg of lichen/mL). Galantamine was used as a positive control.

#### 2.7.2. α-Glucosidase Inhibition Assay

α-glucosidase inhibition assay was performed according to the method reported by Costamagna et al. and Burgos-Edwards et al. [20,21]. Briefly, the reaction mixture contained sodium phosphate buffer (200 mM, pH 6.9), solutions of the extracts at different concentrations prepared in the same buffer and α-glucosidase (0.1 U/L). After 15 min of preincubation at 37 °C, the reaction was started by adding p-nitrophenyl-α-d-glucopyranoside (5 mM) into the wells and incubated for 30 min at 37 °C, and absorbance was measured at 415 nm in a microplate reader (BioTek Instrument, Inc., Winooski, VT, USA). The results were expressed as IC_50_ values (μg of lichen/mL). Acarbose was used as a positive control.

#### 2.7.3. Lipase Inhibition Assay

This assay was performed according to the method reported by McDougall et al. and Picot et al. [22,23]. Porcine pancreatic lipase type II was resuspended in ultrapure water at 20 mg/mL. The substrate 4-nitrophenyl-dodecanoate was prepared with DMSO and ethanol. Briefly, the assay mixture was Tris-HCl buffer (0.1 M, pH 8.5), lipase (10 mg/mL) and solutions of the extracts at different concentrations, the substrate solution (5 mM) was incubated for 15 min at 37 °C and absorbance was measured at 410 nm in a microplate reader (BioTek Instrument, Inc., Winooski, VT, USA). The results were expressed as IC_50_ values (μg of lichen/mL). Orlistat was used as a positive control.

### 2.8. Calculation of ADME Parameters

Osiris Data Warrior toolkits (v 5.5.0) were used to verify the pharmacokinetic properties of compounds present in extracts of *C. gracilis* and *C. chlorophaea* species (usnic acid, thamnolic acid, fumarprotocetraric acid (FP acid), orsellinic acid, atranorin, cetraric acid, squamatic acid and methylorsellinate), compounds reported for the genus *Cladonia* (13-(beta-d-glucosyloxy)docosanoic acid (13D acid)), squaric acid, perlatolic acid and psoromic acid) and the commercial inhibitor of 5-LOX (Zileuton). The molecular descriptors that were calculated are the logarithm of the partition coefficient (cLogP), the number of hydrogen bond donors, the number of hydrogen bond acceptors, the molecular mass of compounds, the topological polar surface area (TPSA), the number of rotable bonds and violations of Lipinski’s rule of five. The absorption percentage (% ABS) was calculated using the following equation [24,25,26]:

% ABS = 109 − (0.345 × TPSA)(1)

### 2.9. Calculation of Risk Toxicity

To calculate the toxicological properties of compounds present in extracts of *C. gracilis* and *C. chlorophaea* species (usnic acid, thamnolic acid, FP acid, orsellinic acid, atranorin, cetraric acid, squamatic acid and methylorsellinate), compounds reported for the genus *Cladonia* (13D acid, squaric acid, perlatolic acid and psoromic acid) and the commercial inhibitor of 5-LOX (Zileuton), the Osiris Data Warrior computational tool was used. The toxicity risks evaluated in each of the molecules were mutagenicity, tumorigenicity, irritation and reproductive effects [25,26].

### 2.10. In Silico Analysis

The crystallographic structure of the enzyme arachidonate 5-lipoxygenase (5-LOX) was obtained from the RCSB PDB (Research Collaboratory for Structural Bioinformatics Protein Data Bank) with the PDB ID code 6N2W. This crystallographic structure is crystallized with the nordihydroguaiaretic acid (NDGA) ligand which was used as a reference to carry out a directed coupling in the catalytic site of the NDGA/6N2W complex [9,25].

Enzyme optimization was carried out using UCSF Chimera software (v1.16, San Francisco, California, USA), where water molecules and ligands were removed from the active site of the crystallographic protein. In the same way, all polar hydrogen atoms were added at pH = 7.4. The appropriate ionization states for basic and acidic amino acid residues were considered [25,27].

#### 2.10.1. Ligand Preparation

The two-dimensional structures of the ligands (usnic acid, thamnolic acid, FP acid, orsellinic acid, atranorin, cetraric acid, squamatic acid, methylorsellinate, 13D acid, squaric acid, perlatolic acid and psoromic acid) were prepared with the ChemDraw 8.0 program (PerkinElmer Informatics, Waltham, MA, USA) and imported into the Avogadro program (https://avogadro.cc, accessed on 20 April 2022) to optimize the geometry using the field function of MMFF94 [25,26]. All compounds were saved as mol2 files for docking studies.

#### 2.10.2. Docking Simulation

The standard procedure for molecular docking was developed using the rigid crystallographic structure of the enzyme arachidonate 5-lipoxygenase (6N2W), nine flexible ligands that did not present any risk of toxicity (thamnolic acid, 13D acid, perlatolic acid, psoromic acid, orsellinic acid, atranorin, cetraric acid, squamatic acid and methylorsellinate) and a commercial 5-LOX inhibitor ligand (zileuton) whose torsion angles were identified (during 10 independent runs per ligand). Targeted molecular docking was performed using the UCSF Chimera program [25,27,28], where the reference inhibitor catalytic pocket (NDGA) for arachidonate 5-lipoxygenase (5-LOX) was used. Polar hydrogens and Gasteiger partial charges were added, and a bounding box was created, whose size was fixed to a cube with sides that were 20 Å in length, using the Autodock Vina tools at UCSF Chimera. The centroid of the selected residue was chosen based on the enzyme’s putative catalytic site (5-LOX), considering its known catalytic amino acids: His367, His372, His550 and Leu673. The coupling and analysis results were visualized using the Discovery Studio Visualizer [25,26]. After docking was complete, the best conformation for hydrogen bonding or π interactions was analyzed, including the free ligand binding energy (delta G, kcal/mol) [25,26,29].

### 2.11. Human Neuroblastoma Cell Line (SH-SY5Y Cells)

SH-SY5Y cells were grown in Dulbecco’s modified Eagle’s medium (DMEM) supplemented with 10% fetal bovine serum (FBS) and 0.5% gentamicin at 37 °C and 5% CO_2_ and 95% air. The confluence between 80 and 90% was obtained.

### 2.12. Cell Treatments

A stock solution (1 mg/mL) of each lichen extract was dissolved in DMSO and phosphate-buffered saline (PBS). SH-SY5Y cells were pretreated with different concentrations of the extracts for 24 h, followed by H_2_O_2_ (1 mM, 1 h). The highest concentration treatment had a final DMSO concentration of less than 0.1%.

### 2.13. Metabolic Activity Measurement

Cell survival and the cytoprotection rate were determined according to the MTT colorimetric assay [30], with some modifications. After exposure to the extract treatments, MTT stock solution (2 mg/mL) was added to the wells and incubated for 1 h; subsequently, DMSO (100 µL) was added, and absorbance was measured at 550 nm with a Spectrostar BMG microplate reader.

### 2.14. Intracellular ROS Production

According to the DCFH-DA assay [31] the intracellular production of ROS was determined. In the 96-well plate, DCFH-DA (24 µL) was added in the DMEM medium (1%) without phenol red (12 mL) for 30 min, and then the medium was aspirated. The most effective concentrations of the lichen extracts were added; after 1 h, hydrogen peroxide (250 mM) was added. Fluorescence was measured with a microplate reader (FLUOstar OPTIMA, BMG Labtech, Ortenberg, Germany) at an excitation/emission wavelength of 485/528 nm.

### 2.15. Statistical Analysis

Data were performed in triplicate and expressed as the mean data with standard deviations (SD) using Microsoft Excel 2019 software (Microsoft Office, Microsoft Corporation, Redmond, WA, USA). Statistical significance between groups was set at *p* < 0.05 and determined by one-way ANOVA with Tukey’s post hoc test using the commercial software GraphPad Prism 8.

## 3. Results and Discussion

### 3.1. UHPLC Chromatographic Analysis of Lichens Extracts

**Figure 2:**
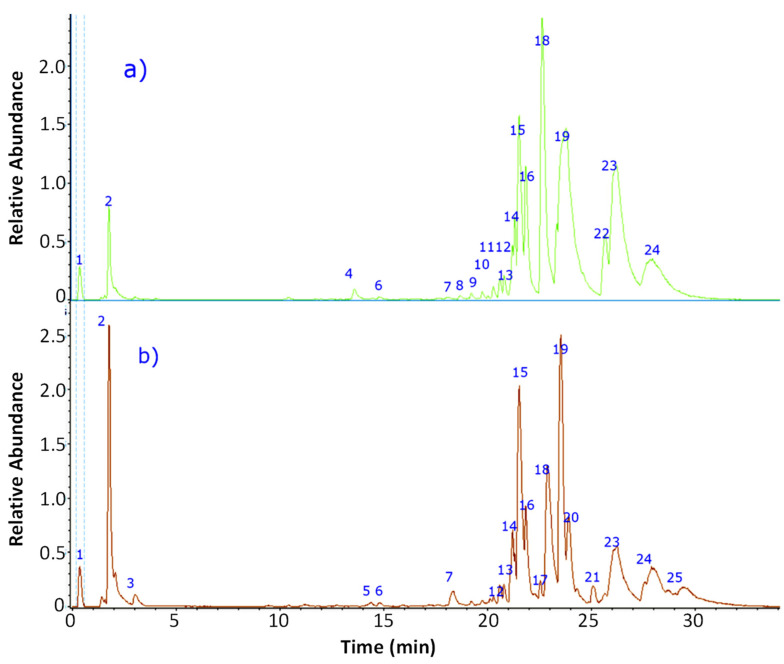
UHPLC-MS Chromatogramans: (**a**) *C. gracilis*; (**b**) *C. chlorophaea*. The numbers of the peaks correspond to the identification indicated in Table 1.

The fingerprinting of the ethanolic extracts of *C. gracilis* and *C. chlorophaea* were obtained by high-resolution mass spectrometric analysis (UHPLC-MS). The negative mode was used for compound identification. In total, nineteen metabolites were detected in each of the species (Figure 2), and their tentative identification included aromatics, organic acids, lipids, dibenzofurans, depsides and depsidones (Table 1).

Aromatic Derivatives

For *C. gracilis* and *C. chlorophaea*: peak 1 corresponded to Na formiate (C_4_H_2_O_4_) as the internal standard. Peak 6 was identified as squamatic acid (C_19_H_17_O_9_), peak 15, with a molecular anion at *m*/*z* 355.0454, was identified as a criptostictic acid derivate (C_18_H_11_O_8_) and peak 16, with a molecular anion at *m*/*z* 471.0569 and diagnostic peaks at *m*/*z* 375.0668, 355.0385, 167.0300 and 943.0975, was identified as fumarprotocetraric acid (C_22_H_15_O_12_). In *C. gracilis*, peak 4 was identified as methylorsellinate (C_9_H_9_O_4_). In *C. chlorophaea*, peak 3 was identified as orsellinic acid (C_8_H_7_O_3_), peak 5, with an [M-H]^-^ ion at *m*/*z* 373.0926, was identified as atranorin (C_19_H_17_O_8_), with diagnostic peaks at *m*/*z* 177.0186 and 163.0934, and peak 20 was identified as cetraric acid (C_20_H_17_O_9_), with diagnostic peaks at *m*/*z* 281.2464 and 211.0166.

Organic Acids

For *C. gracilis* and *C. chlorophaea*: peak 2 was identified as citric acid (C6H7O7).

Lipids

For *C. gracilis* and *C. chlorophaea*: peak 7 was identified as hexahidroxioxohexacosanoic acid (C_26_H_49_O_9_), peak 12, with a molecular anion at *m*/*z* 489.3432, was identified as pentahydroxyoxohexacosanoic acid (C_26_H_49_O_8_), with diagnostic peaks at *m*/*z* 403.3001 and 979.6848, peak 13, with an [M-H]^-^ ion at *m*/*z* 417.3236, was identified as 9,10,12,13-tetrahidroxytricosanoic acid (C_23_H_45_O_6_), with diagnostic peaks at *m*/*z* 235.0538 and 195.0616, and peak 24 was identified as octadeca-9,12,15-trienoic acid (C_18_H_29_O_2_). In *C. gracilis*, peak 8 was identified as 9,10,12,13-tetrahydroxyheneicosanoic acid (C_21_H_41_O_6_), peak 9 was identified as pentahydroxyhexacosanoic acid (C_26_H_51_O_7_), with diagnostic peaks at *m*/*z* 448.3405 and 273.0163, peak 10 was identified as 9,10,12,13-tetrahydroxydocosanoic acid (C_22_H_43_O_6_) and peak 11 was identified as stearin (C_23_H_45_O_7_). In *C. cholorophaea*, peak 21, with a molecular anion at *m*/*z* 277.2173, was identified as linolenic acid (C_18_H_29_O_2_), and peak 25 was detected but not identified.

Dibenzofurans

For *C. gracilis* and *C. chlorophaea*: peak 14 was identified as pseudoplacodiolic acid (C19H19O8), with diagnostic peaks at *m*/*z* 343.0807, 259.0598 and 231.0648, and peak 19 was identified as usnic acid (C18H15O7).

Depsidones

For *C. gracilis* and *C. chlorophaea*: peak 18, with a molecular anion at *m*/*z* 401.0593, was identified as constictic acid or siphulellic acid (C19H14O10).

Depsides

For *C. gracilis* and *C. chlorophaea*: peak 23 was identified as ramaric acid (C18H17O7). In *C. gracilis*, peak 22 was identified as lecanoric acid (C16H13O7). In *C. chlorophaea*, peak 17, with a molecular anion at *m*/*z* 419.0614, was identified as thamnolic acid (C19H15O11), with diagnostic peaks at *m*/*z* 211.0201 and 317.0592.

The group of compounds identified in the ethanolic extracts of the lichens species *C. chlorophaea* and *C. gracilis* form a chemical fingerprint related to the recent metabolomic reports of other Antarctic lichens such as *C. metacorallifera* with 26 compounds [32], *Usnea antarctica* with 21 compounds [33], *Himantormia lugubris* with 28 compounds [8], *Lecania brailmontt*i with 18 compounds, *Pseudephebe pubescens* with 18 compounds and *Sphaero- phorus globosus* with 14 compounds [26]; some of them, as fumarprotocetraric, lecanoric, squamatic and usnic acids, represent typical compounds in foliose lichens that participate in the biochemical mechanisms for the adaptation of lichens to the habitat [34]. Likewise, the chemical study of extracts is the basis for the detection of new compounds with biological potential, as in the case of the isolation of three p-terphenyls and two fumarprotocetraric acid lactones in the Antarctic lichens *Stereocaulon alpinum* [35] and *C. metacora- llifera* [32], respectively.

### 3.2. Total Phenolic Contents and Antioxidant Activity

The results corresponding to phenolic content and antioxidant activity measured by the different colorimetric assays are detailed in Table 2. The ethanolic extract of *C. chlorophaea* showed six-times-higher phenolic content compared to *C. gracilis*, with 330.276 ± 0.006 and 53.563 ± 0.004 mg gallic acid/100 g of extract, respectively. For the antioxidant activity, in the FRAP and ORAC assays, the *C. chlorophaea* extract presented better results compared to the *C. gracilis* extract; as for the DPPH assay, both extracts presented a high IC_50_, it being higher for the *C. chlorophaea* extract.

The ethanolic extracts of *C. chlorophaea* and *C. gracilis* showed phenolic contents and antioxidant activities similar to those reported in the methanolic, acetonic, chloroformic and hydroalcoholic extracts of other species of *Cladonia*; however, there are variable cases such as the sample of *C. foliaceae*, where the methanolic extract did not show activity in the DPPH assay, and the percentage of phenolic compounds was 1.7% [36]. In *C. furcata*, an IC_50_ >1000 and 471.3 ± 35.4 µg/mL were reported for DPPH in the methanolic and acetonic extracts, respectively [37]; in other works, an IC_50_ in DPPH of 967.97 ± 2.35 µg/mL and a phenol content of 28.00 ± 1.065 mg GAE/g [38] were reported, as well as a 44.83% effect on DPPH activity and a phenolic content of 12.95 ± 1.065 µg pyrocatechol/g [39].

In other cases, such as *C. pyxidata* and *C. rangiferina*, for the acetone extract, the ra- dical scavenging activity was 461.72 ± 2.93 and 987.64 ± 2.02 µg/mL, respectively, and the phenol content was 35.35 ± 1.013 and 22.00 ± 1.065 mg GAE/g, respectively [38]. In *C. amaurocraea* and *C. mediterranea*, the content of phenolic compounds in acetonic, ethanolic and methanolic extracts was considerably low, with values between 0.005 to 0.174 and 0.006 to 0.009 mg GAE/g, respectively, compared to the amount of proteins and carbohydrates [40]. In *C. clathrata* with the hydroalcoholic extract, a total phenol concentration of 371.8 ± 57.3 mg GAE/g and a DPPH of 69.25 ± 0.65 µg/mL were found [41], similar to *C. verticillata*, with phenol contents of 101. 37 ± 3.31 and 48.61 ±0.87 mg GAE/g in the acetonic and methanolic extract, respectively, and the DPPH assay, with 33.09 ± 0.84 and 17.25 ± 2.98 µg/mL for the acetonic and methanolic extract, respectively [42].

In other species such as *C. rangiformis*, the reported phenolic contents are low, with 0.528 ± 0.007 and 0.103 ± 0.012 mg GAE/g in the chloroform and methanol extracts, respectively [43]. In a work with a majority of compounds from 15 species of the genus *Cladonia* [44], they report, for DPPH radicals, an IC_50_ of 207.8 ± 31.6, 320.0 ± 48.1, 145.4 ± 17.3, 394. 1 ± 55.2, 952.4 ± 85.6, 153.8 ± 21.5 and 252.4 ± 30.3 µg/mL in atranorin, barbatic, fumarprotocetraric, perlatolic, squamatic, thamnolic and usnic acids, respectively [7]. For specimens of *Cladonia* sp., a DPPH of 38.5 ± 3.8 µg/mL has been reported [45]. In general, Antarctic lichen extracts show significant antioxidant activities that enhance their adaptation to extreme environmental conditions [46].

In comparison with other Antarctic lichens, the ethanolic extracts of the two *Cladonia* species performed better than the ethanolic extracts of *Himantormia lugubris* [8] and *Lecania brialmontii, Pseudephebe pubescens* and *Sphaerophorus globosus* [26], confirming the variable antioxidant potential of species from the polar territories.

### 3.3. Enzymatic Inhibitory Activity

The values obtained with the ethanolic extracts of the two lichen species are shown in Table 3. As for the cholinesterases’ inhibition activity, the results of the two extracts were potent and close to the values recorded for the standard compound galantamine; for AChE, the extract of *C. chlorophaea* showed a better IC_50_ value compared to *C. gracilis*, with 4.204 ± 0.061 and 6.211 ± 0.055, respectively; likewise, for BChE, the extract of *C. chlorophaea* showed better results compared to *C. gracilis*, with 5.938 ± 0.069 and 9.105 ± 0.065, respectively. As for digestive enzyme activity, the *C. gracilis* and *C. chlorophaea* extracts evidenced higher α-glucosidase inhibition (91.323 ± 0.010 and 108.590 ± 0. 006, respectively) against the reference compound acarbose; for pancreatic lipase, both extracts showed low inhibitory activity (*C. chlorophaea* with 125.310 ± 0.049 and *C. gracilis* with 345.135 ± 0.050) compared to the positive control Orlistat.

The anticholinergic activity of ethanolic extracts of *C. chlorophaea* and *C. gracilis* was more effective against the ethanolic extract of Antarctic species such as *H. lugubris*, with an IC_50_ of 12.38 ± 0.09 and 31.54 µg/mL in AChE and BChE, respectively [8]; *L. brialmontii, P. pubescens* and *S. globosus* presented similar values in AChE, with 3.949 ± 0.04, 2.805 ± 0.07 and 10.422 ± 0.08 µg/mL, respectively, and in BChE, with 4.476 ± 0.06, 8.828 ± 0.08 and 6.785 ± 0.04 µg/mL, respectively [26]. With the compound buruloquinone isolated from *C. macilenta*, an AChE inhibition of 27.1 µg/mL has been reported [47], demonstrating the inhibitory potential of major components isolated from lichen extracts; in *C. uncialis*, values for AChE between 0.1 ± 0.0 and 7.9 ± 0.0 µg/mL and for BChE between 9.5 ± 0.4 and 85.9 ± 0.2 µg/mL were reported based on reference compounds [48]. Regarding α-glucosidase, the values reported for other lichenic species are lower, with IC_50_ >3 and 0.6 ± 0.0, 17.5 and 10.7–17.6 µg/mL for *Xanthoria elegans* and *X. parietina* [49], *Parmotrema dilatatatum* [50] and compounds isolated from *P. tsavoense*, respectively [51]. In the pancreatic lipase, *X. elegans, X. parietina* and *X. candelaria* reported lower values, with an IC_50_ of 79 ± 5.0, 68 ± 5.0 and 55 ± 3.0 µg/mL, respectively [49].

### 3.4. In Silico Anti-Inflammatory Effect of 5-LOX

#### 3.4.1. Prediction of Pharmacokinetic Properties—ADME

The analysis of the pharmacokinetic properties of the compounds present in the species of the genus *Cladonia* (usnic acid, thamnolic acid, 13D acid, squaric acid, perlatolic acid, FP acid, psoromic acid, orsellinic acid, atranorin, cetraric acid, squamatic acid and methylorsellinate) was calculated with the Osiris Data Warrior computational tool, where the results of these compounds were compared with the pharmacokinetic properties of a commercial 5-LOX inhibitor (Zileuton) (Table 4). Drugs that are administered orally must meet certain parameters, which are known as the Lipinski rules [25,26]. These rules allow for the evaluation and monitoring of drugs based on biological and pharmacological function. The parameters encompassed by Lipinski’s rules are that the drug must have a molecular weight of less than 500 g/mol; cLogP must have a value less than 5; in the molecular structure there must be a maximum of five hydrogen donor sites; the compound must have 10 or fewer hydrogen bond receptor sites; finally, the compound must have less than 10 rotatable bonds in its chemical structure [25,26]. The molecules that had more than one violation of the Lipinski rules were the compound 13D acid (three violations) and perlatolic acid (two violations). These analyses show that these compounds may have a low bioavailability and pharmacokinetic properties, which make it difficult for them to be administered orally. All compounds were compared with the commercial 5-LOX inhibitor (zileuton), showing that usnic acid, squaric acid, psoromic acid, orsellinic acid, atranorin, cetraric acid, squamatic acid and methylorsellinate presented behaviors similar to zileuton, which did not present any violation of the Lipinski rules. Additionally, we observed that the compounds thamnolic acid and FP acid only presented a violation of the Lipinski rules, which are in the limit to be accepted as possible inhibitors of 5-LOX and to be administered orally; however, in silico and in vitro analyses are required to enrich and further support the in silico analysis. The bioavailability of compounds presents in *Cladonia* is also assessed by topological polar surface area analysis (TPSA) [24,25,26]. This parameter is highly related to the passive molecular transport that a drug can undergo through cell membranes and can predict the transport properties that the compounds can present, as well as their bioavailability. With the TPSA parameter, it was possible to predict the absorption percentage of each of the compounds, comparing them with the commercial inhibitor Zileuton [24]. The compounds that presented a higher percentage of absorption compared to zileuton (76.30%) were squaric acid (83.26%), orselinic acid (82.17%) and methylorselinate (85.97%).

#### 3.4.2. Toxicity Prediction

**Figure 3:**
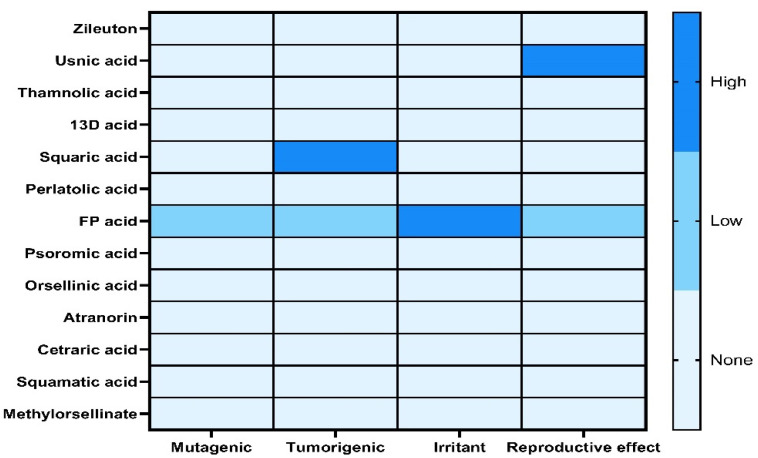
The heat map representation of the toxicity risk of compounds present in the species of the genus *Cladonia* compared to the 5-LOX redox inhibitor (Zileuton) was realized with GraphPad Prism v.7 (GraphPad; La Jolla, CA, USA).

To calculate the toxicity risk parameters that the compounds in the species of the genus *Cladonia* could present, the Osiris Data Warrior computational tool was used [25,26]. This computational tool allows for the evaluation of the molecules according to their chemical structure and the possible molecular fragments that can cause some toxicological risk. The toxicity risk parameters that were calculated are mutagenicity, tumorigenicity, irritation and reproductive toxicity [25,26]. The results are displayed using a blue color scale, where the most intense blue color indicates that the compound exhibits a high risk of toxicity, an intermediate blue color indicates that the compound exhibits an intermediate toxicity risk and a weaker blue color indicates that the compound does not present any toxicity risk (Figure 3). The compounds that presented risks of toxicity were usnic acid, squaric acid and FP acid. Usnic acid presented a high risk of toxicity in the reproductive effect because it presented a fragment in its chemical structure that confers this toxicity effect. The squaric acid exhibited a high probability of presenting tumorigenic properties because the entire chemical structure of squaric acid gives it these risks and toxicological properties. The FP acid compound presented various toxicological risks because its chemical fragmentation forms unstable chemical structures that give it these toxicological properties. FP acid exhibited five fragments with a high risk of an irritant effect; likewise, low risks of mutagenicity, tumorigenicity and toxicity in the reproductive effect were observed (Table 5).

#### 3.4.3. Evaluation of the Docking 5-LOX Inhibition

The directed coupling of 9 of the 12 compounds present in the species of the genus *Cladonia* was performed with the enzyme 5-LOX (PDB ID: 6N2W) to see the possible interactions with the catalytic site. The 5-LOX catalytic site is composed of a tetrad of catalytic residues (His367, His372, His550 and Leu673) that, together with the iron atom, coordinate for its catalysis. Ten conformations were tested in each of the compounds to be evaluated, selecting the best conformation that presented a better binding affinity (∆G, kcal/mol) and comparing them with a commercial 5-LOX inhibitor (Zileuton) [25,26].

The results in Table 6 showed that the one with the best binding affinity for the 5-LOX enzyme was psoromic acid (−8.30 kcal/mol); furthermore, the binding energy shown by this compound was very close compared to the commercial inhibitor zileuton (−8.70 kcal/mol). The compounds thamnolic acid, perlatolic acid, atranorin, cetraric acid and squamatic acid exhibited similar behaviors in terms of binding affinity (−7.40, −7.10, −7.20, −7.60 and −7.60 kcal/mol, respectively); however, they were inferior to zileuton and psoromic acid. The compounds that presented a lower binding affinity were 13D acid (−5.60 kcal/mol), orsellinic acid (−5.60 kcal/mol) and methylorsellinate (−5.40 kcal/mol); however, these compounds showed specific interactions with the catalytic site of 5-LOX.

**Figure 4:**
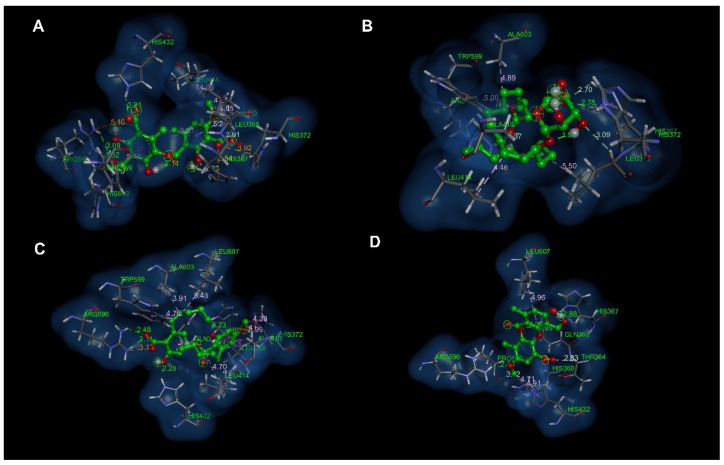
Molecular docking of compounds present in the species of *Cladonia*. (**A**) Higher affinity conformation of thamnolic acid with 5-LOX; (**B**) Higher affinity conformation of 13D acid with 5-LOX; (**C**) Higher affinity conformation of Perlatolic acid with 5-LOX; (**D**) Conformation of a higher affinity of psoromic acid with 5-LOX.

**Figure 5:**
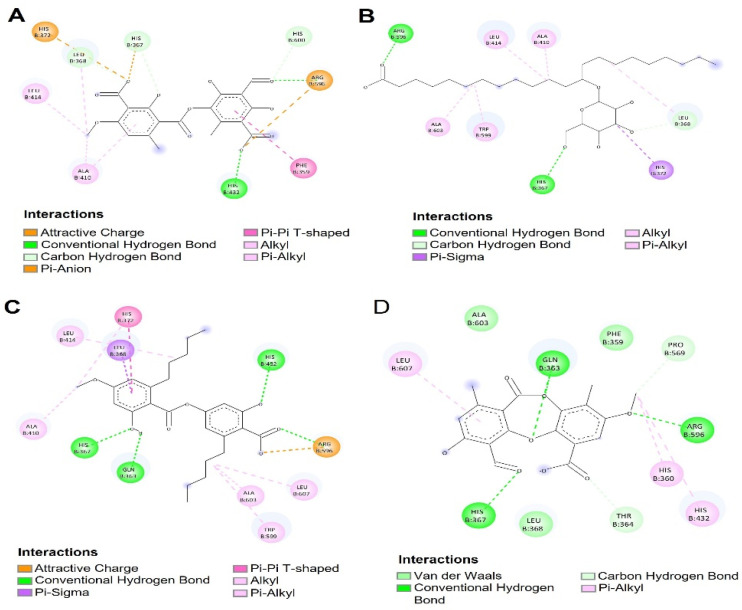
Molecular interactions of the compounds present in the species of *Cladonia*. (**A**) Molecular interactions of Thamnolic acid with the 5-LOX catalytic pocket; (**B**) Molecular interactions of 13D acid with the 5-LOX catalytic pocket; (**C**) Molecular interactions of Perlatolic acid with the catalytic pocket of 5-LOX; (**D**) Molecular interactions of Psoromic acid with the 5-LOX catalytic pocket.

Figure 4 and Figure 5 show the molecular interactions and distances shown for thamnolic acid, 13D acid, perlatolic acid and psoromic acid with the 5-LOX protein. The thamnolic acid showed a strong interaction and hydrogen bond, with a residue His432 distance of 2.21 Å (Figure 4A and Figure 5A). The carboxylate present in one of the aromatic rings of the thamnolic acid presented strong interactions of the charge attraction type with the following residues: an Arg596 distance of 1.82 Å and a His372 distance of 3.92 Å (Figure 4A and Figure 5A); the latter is highly involved in the catalytic site for the inhibition of the 5-LOX enzyme. Carbon hydrogen bond type interactions were observed, with a His367 distance of 2.83 Å, a Leu368 distance of 2.91 Å and a His600 distance of 2.87 Å, where the His367 residue is directly involved in the inhibition of the 5-LOX enzyme.

The 13D acid presented two strong hydrogen bond type interactions (Figure 4B and Figure 5B) with the residues Arg596 (distance 1.87 Å) and His367 (distance 2.78 Å), which allowed it to stabilize within the catalytic pocket. Pi-sigma interactions (Figure 4B and Figure 5B) with His372 (distance 3.47 Å) were observed between the imidazole chain of His372 and the carbon of the tetrahydropyran group of 13D acid. Alkyl interactions (Figure 4B and Figure 5B) were observed between the methylenes of the alkyl chain of 13D acid and the residues Ala410 (distance 4.37 Å), Leu414 (distance 4.46 Å), Trp599 (distance 5.00 Å) and Ala603 (distance 4.89 Å).

Figure 4C and Figure 5C show the molecular interactions between the catalytic site of 5-LOX and perlatolic acid. In this figure, three hydrogen bonding interactions were presented with the residues Gln363 (distance 1.93 Å), His367 (distance 2.23 Å) and His432 (distance 2.28 Å). Charge attraction interactions were exhibited between the residue Arg596 (distance 3.13 Å), and it was also observed that this residue also presented hydrogen bond type interactions (distance 2.11 and 2.48 Å) with the carboxylate group of perlatolic acid (Figure 4C and Figure 5C).

The psoromic acid had hydrogen bonding interactions with the residues Gln363 (distance 1.99 Å), His367 (distance 2.88 Å) and Arg596 (distance 2.46 Å), which maintained the stability of the psoromic acid at the 5-LOX catalytic site (Figure 4D and Figure 5D). Carbon hydrogen interactions occurred between the Pro569 residue (distance 3.42 Å) and the methoxyl group of one of the aromatic rings of psoromic acid; the other carbon hydrogen interaction was shown with the residue Thr364 (distance 2.83 Å) and the carboxylate group of the aromatic ring of psoromic acid (Figure 4D and Figure 5D).

**Figure 6:**
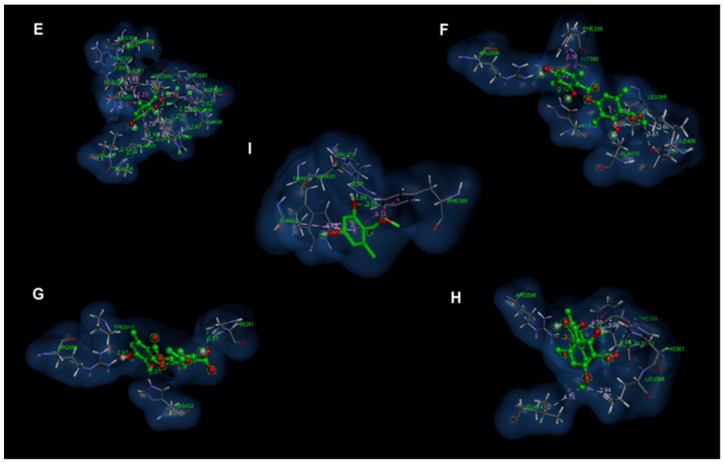
Molecular docking of compounds present in the species of *Cladonia*. (**E**) Conformation of a higher affinity of orsellinic acid with 5-LOX; (**F**) Conformation of a higher affinity of atranorin with 5-LOX; (**G**) Conformation of a higher affinity of cetraric acid with 5-LOX; (**H**) Higher affinity conformation of squamatic acid with 5-LOX; (**I**) Higher affinity conformation of methylorsellinate with 5-LOX.

**Figure 7:**
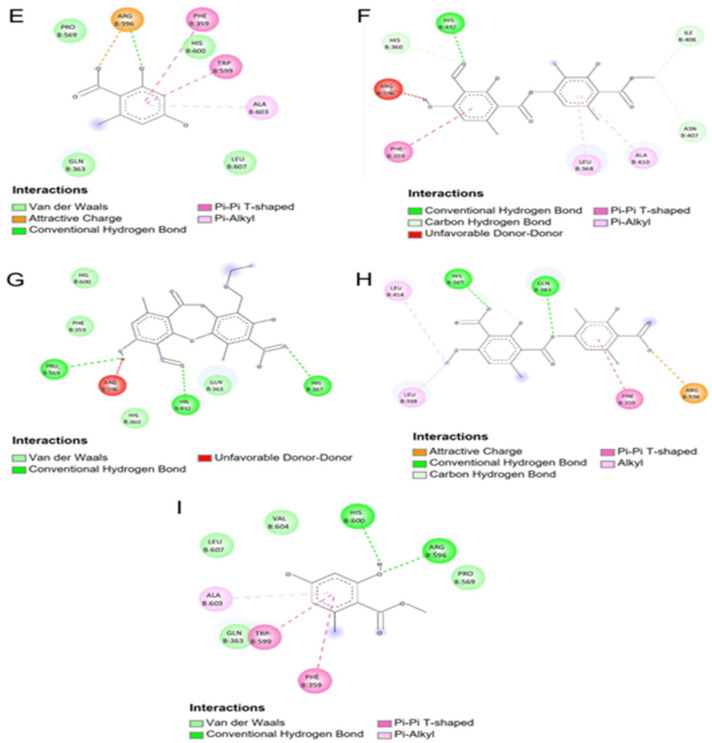
Molecular interactions of compounds present in the species of *Cladonia*. (**E**) Molecular interactions of orsellinic acid with the catalytic pocket of 5-LOX; (**F**) Molecular interactions of atranorin with the 5-LOX catalytic pocket; (**G**) Molecular interactions of cetraric acid with the catalytic pocket of 5-LOX; (**H**) Molecular interactions of squamatic acid with the 5-LOX catalytic pocket; (**I**) Molecular interactions of methylorsellinate with the 5-LOX catalytic pocket.

As for Figure 6 and Figure 7, Figure 6E and Figure 7E show the molecular interactions between the catalytic site of 5-LOX and orsellinic acid; a hydrogen bridge type bond and a charge attraction interaction were shown with the Arg596 residue (distances of 1.87 Å and 3.13 Å, respectively). Two π-π T-shaped interactions were presented between the pi electrons of the aromatic ring of the orsellinic acid and the π electrons of the residues Phe359 (distance 5.23 Å) and Trp599 (distance 5.22 Å). The Ala603 residue had a π-alkyl type interaction between the π electrons of the aromatic ring of the orsellinic acid and the methyl group of the Ala603 residue (distance 4.70 Å).

Atranorin (Figure 6F and Figure 7F) and cetraric acid (Figure 6G and Figure 7G) were the compounds that presented unfavorable donor–donor interactions and were precisely present in the hydroxyl group present in the aromatic ring in the structures of the compounds and between the residue Arg596 (distances of 1.17 Å and 1.18 Å); however, it was observed that cetraric acid presented strong hydrogen bonding interactions with the residues His432 (distance 2.01 Å), His367 (distance 2.51 Å) and Pro569 (distance 2.97 Å).

In Figure 6H and Figure 7H, two hydrogen bond interactions are observed, which allowed for the stability of the squamatic acid in the catalytic site; these interactions occurred with His367 (distance 2.66 Å) and Gln363 (distance 2.12 Å) residues. Charge attraction interactions were observed between the carboxylate functional group of the squamatic acid and the amine group of the Arg596 residue (distance 3.31 Å). Figure 6I and Figure 7I show the behavior of methylorsellinate at the catalytic site of the 5-LOX enzyme. In these images, we can observe the interactions that this compound presented with the catalytic site. The methylorsellinate presented hydrogen bonding interactions between the hydroxyl group of the aromatic ring and the residues Arg596 (distance 1.94 Å) and His600 (distance 2.08 Å). Additionally, π-π T-shaped interactions were presented between the π electrons of the aromatic ring of methylorsellinate and the π electrons of the aromatic rings of the residues Phe359 (distance 5.11 Å) and Trp599 (distance 5.38 Å).

### 3.5. Cytoprotective Activity

#### 3.5.1. Effect of *Cladonia* Extracts on Cell Viability and Survival under Oxidative Stress Induced by H_2_O_2_

**Figure 8:**
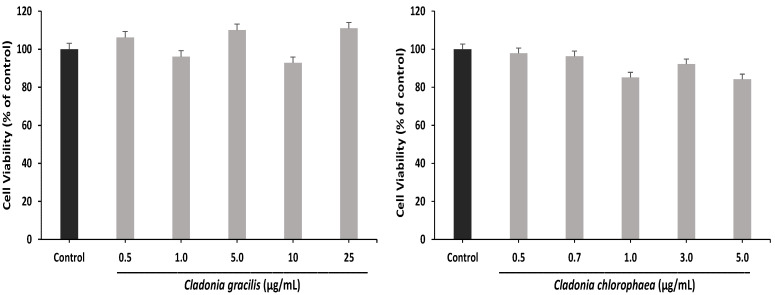
Effect of *C. gracilis* and *C. chlorophaea* extracts on viability in the SH-SY5Y cell line. The values represent the means ± SD of three replicates (n = 3).

First, the effect of the ethanolic extracts of *C. chlorophaea* and *C. gracilis* on the viability of the SH-SY5Y cell line was determined by quantification with the MTT (3-[4,5-dimethylthiazol-2-yl]-2,5 diphenyl tetrazolium bromide) assay. According to previous cytotoxicity assays, five independent concentrations were determined for each extract: *C. gracilis* with 0.5, 1.0, 5.0, 10 and 25 µg/mL and *C. chlorophaea* with 0.5, 0.7, 1.0, 3.0 and 5.0 µg/mL. The *C. gracilis* extract promoted over 90% cell viability at all the concentrations evaluated; on the other hand, the *C. chlorophaea* extract showed optimal cell viability, but at 1.0 and 5.0 µg/mL, a slight reduction in viability was found (85% and 84%, respectively) (Figure 8).

**Figure 9:**
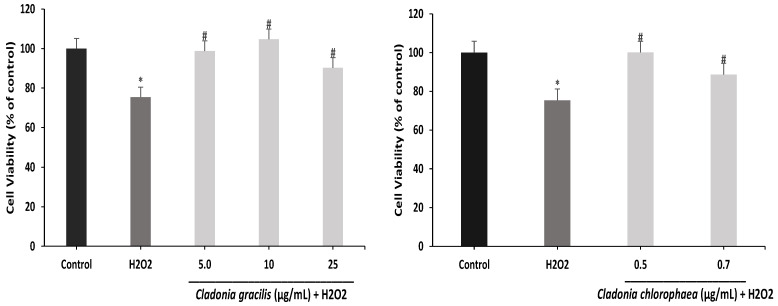
Effect of *C. gracilis* and *C. chlorophaea* extracts on cytoprotection in an H_2_O_2_-induced oxidative stress model. The values represent the means ± SD of three replicates (n = 3). Values marked with * and # are statistically different using the Tukey test at a 0.05 level of significance (*p* ˂ 0.05) versus the control and versus H_2_O_2_, respectively.

Subsequently, the most optimal concentrations of cell viability in the two extracts were selected to determine the protective effector against H_2_O_2_-induced oxidative stress: *C. gracilis* at 5.0, 10 and 25 µg/mL and *C. chlorophaea* at 0.5 and 0.7 µg/mL. According to Figure 9, H_2_O_2_ treatment decreased the viability of SH-SY5Y cells by 75% compared to control cells. On the other hand, the two extracts promoted cell survival compared to H_2_O_2_ treatment; thus, for *C. gracilis*, the increase in cell viability was 98.7%, 104.7% and 90.2% at 5.0, 10 and 25 µg/mL, respectively, and for *C. chlorophaea*, the increase was 100% and 88% at 0.5 and 0.7 µg/mL, respectively.

#### 3.5.2. Effect of *Cladonia* Extracts on the Reduction in ROS Production in an H_2_O_2_-Induced Oxidative Stress Model

**Figure 10:**
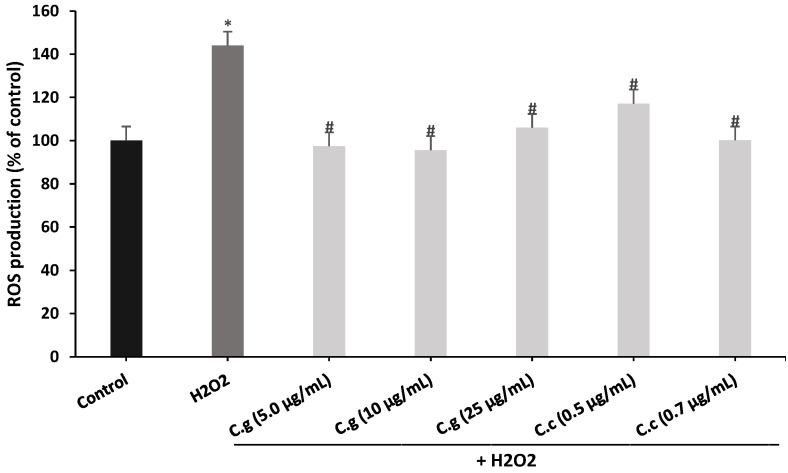
Effect of *C. gracilis* and *C. chlorophaea* extracts on intracellular ROS production. The values represent the means ± SD of three replicates (n = 3). Values marked with * and # are statistically different using the Tukey test at a 0.05 level of significance (*p* ˂ 0.05) versus the control and versus H_2_O_2_, respectively. C.g: *C. gracilis*; C.c: *C. chlorophaea*.

In this experiment, the concentrations of the extracts used in the previous assay were used to deepen their cytoprotective potential. According to Figure 10, a significant increase in intracellular ROS production of 143.9% was evidenced in the treatment of SH-SY5Y cells with H_2_O_2_ versus the control treatment (100%). In parallel, treatments with ethanolic extracts of *Cladonia* species significantly reduced ROS production; thus, *C. gracilis* showed a reduction of 46.5%, 48.3%, and 38% at 5.0, 10 and 25 µg/mL, respectively, versus treatment with H_2_O_2_, and *C. chlorophaea* showed a reduction of 26.8% and 43.8% at 0.5 and 0.7 µg/mL, respectively, versus treatment with H_2_O_2_.

In other lichen species such as *Dactylina arctica*, *Nephromopsis stracheyi*, *Tuckermannopsis americana* and *Vulpicida pinastri* from cetrarioid clade, ideal viability ranges have been reported with methanolic extracts at concentrations of 5 to 10 µg/mL, along with a reduction of the same between 25 and 50 µg/mL; likewise, cell protection against H_2_O_2_ was e- videnced, especially in *D. artica* and *V. pinastri*, with a viability percentage of 76.8% at 10 µg/mL and 78.9% at 5 µg/mL, respectively. In addition, for *D. arctica*, a reduction in ROS production of 42.4% was reported at 10 µg/mL compared to H_2_O_2_ treatment [12], which is similar to that obtained with *C. gracilis* species at concentrations of 5 and 10 µg/mL. This effective cytoprotective activity is triggered by major compounds in the extracts such as usnic acid and fumarprotetratric acid, which report high antioxidant properties that promote the reduction of oxidative damage in different cell lines [52]. In other studies, isolated compounds such as evernic acid, which are abundantly present in lichens of the genera *Ramalina*, *Evernia* and *Hypogymnia*, report a cell viability >50% using primary culture neurons [11]; biruloquinone, isolated from *C. macilenta* [47], showed a protective e- ffect against H_2_O_2_-induced oxidative stress >80% at a concentration of 25 µg/mL and a reduction in ROS production at 25 and 12.5 µg/mL, which are similar to the values recorded for *C. gracilis* at working concentrations. In parallel, the viability assays can be contrasted with cytotoxicity analyses on cancer cell lines that show the promising use of compounds isolated from lichens for pharmacological treatment, such as the case of (-)-usnic, fumarprotocetraric and 9′(O-methyl)protocetraric acids from *C. convoluta* [53] and di- ffractaic acid, vicanicin, lobaric acid, variolaric acid, protolichesterinic acid and usnic acid from *Protousnea magellanica*, *Psoroma pallidum*, *Stereocaulon alpinum*, *Ochrolechia deceptionis*, *Cornicularia aculeata* and *C. lepidophora*, respectively [54].

## 4. Conclusions

This work contributed to the understanding of the metabolomics of the species of the genus *Cladonia*, *C. chlorophaea* and *C. gracilis*, which are present in the Antarctic territory, reporting the identification of 19 compounds for each one by UHCPL-ESI-QTOF-MS ana- lysis, including aromatics, organic acids, lipids, dibenzofurans, depsides and depsidones, which are typical of lichenized fungi. *C. chlorophaea* and *C. gracilis* species presented va- riable but significant results in terms of the phenolic compound content, FRAP ORAC antioxidant assays, cholinesterases (AChE and BChE) and α-glucosidase inhibition and moderate values in terms of the DPPH antioxidant assay and pancreatic lipase inhibition. On the other hand, in an anti-inflammatory potential approach, molecular docking evidenced significant binding affinities of some compounds for the 5-LOX enzyme, together with outstanding pharmacokinetic properties that compromise their good bioavailability. Likewise, the two extracts of the *Cladonia* species promoted protective activity in the human neuroblastoma cell line against cytotoxicity effects generated by hydrogen peroxide. These species represent a biological resource of scientific interest for the prevention and treatment of central nervous system pathologies, inflammatory disorders and metabolic alterations. Future research could address the isolation and study of bioactive compounds of both species and their molecular mechanisms of action.

## Figures and Tables

**Table 1:** Identification of metabolites in *C. gracilis* and *C. chlorophaea* by UHPLC-ESI-QTOF-MS.

Peak	Tentative Identification	[M − H]^−^	Retention Time (min)	Theoretical Mass (*m*/*z*)	Measured Mass (*m*/*z*)	Accuracy (ppm)	Metabolite Type	MS Ions (ppm)	Lichens
1	Na formiate (internal standard)	C_4_H_2_O_4_	1.04	112.9829	112.9856	3.1	A	-	CG, CC
2	Citric acid	C_6_H_7_O_7_	1.78	191.0192	191.0184	4.2	OA	111.0074	CG, CC
3	Orsellinic acid	C_8_H_7_O_3_	2.2	151.0465	151.0403	5.0	A	-	CC
4	Methylorsellinate	C_9_H_9_O_4_	12.8	181.0506	181.0478	−15.4	A	-	CG
5	Atranorin *	C_19_H_17_O_8_	14.3	373.0926	373.0928	0.19	A	177.0186, 163.0934	CC
6	Squamatic acid	C_19_H_17_O_9_	14.8	389.0877	389.0878	0.45	A	181.05000	CG, CC
7	Hexahidroxioxohexacosanoic acid	C_26_H_49_O_9_	18.2	505.3338	505.3356	3.5	L	431.3015, 375.2722	CG, CC
8	9,10,12,13-Tetrahydroxyheneicosanoic acid	C_21_H_41_O_6_	18.8	389.2903	389.2841	−15.9	L	-	CG
9	Pentahydroxyhexacosanoic acid	C_26_H_51_O_7_	19.2	475.3315	475.3276	8.1	L	448.3405, 273.0163	CG
10	9,10,12,13-Tetrahydroxydocosanoic acid	C_22_H_43_O_6_	19.8	403.3060	403.3000	−14.8	L	-	CG
11	Stearin	C_23_H_45_O_7_	20.2	433.3117	433.3144	−6.0	L	277.2144	CG
12	Pentahydroxyoxohexacosanoic acid	C_26_H_49_O_8_	20.7	489.3432	489.3403	−5.9	L	403. 3001, 979.6848 (2M-H)	CG, CC
13	9,10,12,13-Tetrahidroxytricosanoic acid	C_23_H_45_O_6_	21.1	417.3236	417.3184	−12.4	L	235.0538, 195.0616	CG, CC
14	Pseudoplacodiolic acid or Placodiolic acid	C_19_H_19_O_8_	21.3	375.1080	375.1070	2.7	DBF	343.0807; 259.0598; 231.0648	CG, CC
15	Criptostictic acid derivate	C_18_H_11_O_8_	21.7	355.0454	355.0384	−19.7	A	-	CG, CC
16	Fumarprotocetraric acid	C_22_H_15_O_12_	21.9	471.0569	471.0476	−9.6	A	375.0668, 355.0385, 167.0300, 943.0975 (2M − H)	CG, CC
17	Thamnolic acid	C_19_H_15_O_11_	22.1	419.0614	419.0586	−6.6	d	211.0201, 317.0592	CC
18	Constictic acid or siphulellic acid	C_19_H_14_O_10_	23.1	401.0593	401.0797	50.8	D	-	CG, CC
19	Usnic acid *	C_18_H_15_O_7_	23.5	343.0823	343.0859	−2.91	DBF	-	CG, CC
20	Cetraric acid	C_20_H_17_O_9_	24.1	401.0878	401.0864	−3.4	A	281.2464, 211.0166	CC
21	Linolenic acid	C_18_H_29_O_2_	25.1	277.2173	277.2121	−18.0	L	183.0182	CC
22	Lecanoric acid	C_16_H_13_O_7_	25.3	317.0666	317.0632	−10.7	d	167.034	CG
23	Ramaric acid	C_18_H_17_O_7_	26.1	345.1038	345.1025	−3.7	d	295.1953	CG, CC
24	Octadeca-9,12,15-trienoic acid	C_18_H_29_O_2_	28.0	277.2203	277.2099	−3.2	L	-	CG, CC
25	Unknown	C_27_H_43_O_4_	28.5	431.3228	431.3166	14.3	L	-	CC

* Identified by spiking experiments with an authentic standard compound. A = aromatic; OA = organic acid; L = lipid; DBF = dibenzofuran; D = depsidone; d = depside. CG: *Cladonia gracilis*; CC: *Cladonia chlorophaea*.

**Table 2:** Total phenolic content (TPC) and antioxidant activity (FRAP; ORAC; DPPH) of *C. gracilis* and *C. chlorophaea*.

Assay	TPC (mg GAE/g)	FRAP (µmol Trolox/g)	ORAC (µmol Trolox/g)	DPPHIC50 (µg/mL)
*C. gracilis*	53.563 ± 0.004 *	41.028 ± 0.004 *	223.088 ± 0.761 *	296.737 ± 0.021 *
*C. chlorophaea*	330.276 ± 0.006 *	142.762 ± 0.002 *	271.483 ± 0.920 *	437.85 ± 0.022 *
Gallic acid ^#^	-	-	-	2.24 ± 0.04

The values represent the means ± SD of three replicates (n = 3). Values marked with * are statistically different using the Tukey test at a 0.05 level of significance (*p* ˂ 0.05). ^#^ Positive control.

**Table 3:** Enzyme inhibitory activity of *C. gracilis* and *C. chlorophaea* extracts.

Assay	AChE IC_50_ (µg/mL)	BChE IC_50_ (µg/mL)	α-Glucosidase IC_50_ (µg/mL)	Pancreatic Lipase IC_50_ (µg/mL)
*C. gracilis*	6.211 ± 0.055 *	9.105 ± 0.065 *	91.323 ± 0.010 *	345.135 ± 0.050 *
*C. chlorophaea*	4.204 ± 0.061 *	5.938 ± 0.069 *	108.590 ± 0.006 *	125.310 ± 0.049 *
Galantamine ^#^	0.266 ± 0.029 *	3.824 ± 0.024 *	-	-
Orlistat^® #^	-	-	-	1.9 ± 0.077
Acarbose ^#^	-	-	192.8 ± 0.004	

The values represent the means ± SD of three replicates (n = 3). Values marked with * are statistically different using the Tukey test at a 0.05 level of significance (*p* ˂ 0.05). ^#^ Positive control. AChE, acetylcholinesterase; BChE, butyrylcholinesterase.

**Table 4:** Pharmacokinetic properties of compounds present in the species of the genus *Cladonia* in comparison with the commercial 5-LOX redox inhibitor (Zileuton) obtained from Osiris Data Warrior and Molinspiration software (v 5.5.0).

Compound	%ABS ^a^	TPSA (Å^2^) ^b^	MW ^c^	cLogP ^d^	HBD ^e^	HBA ^f^	*n*-ROTB ^g^	Violation of Lipinski’s Rule
Rule	-	-	<500	≤5	≤5	≤10	≤10	≤1
Usnic acid	68.30	117.97	344.32	0.91	2	7	2	0
Thamnolic acid	44.17	187.89	420.33	1.57	5	11	7	1
13D acid	61.85	136.68	518.73	5.93	5	8	23	3
Squaric acid	83.26	74.60	114.06	−1.33	2	4	0	0
Perlatolic acid	69.91	113.29	444.52	6.06	3	7	13	2
FP acid	42.12	193.86	472.36	1.86	4	12	7	1
Psoromic acid	67.82	119.36	358.30	2.87	2	8	3	0
Orsellinic acid	82.17	77.76	168.15	0.79	3	4	1	0
Atranorin	64.03	130.36	374.34	2.93	3	8	6	0
Cetraric acid	60.84	139.59	402.35	2.83	3	9	5	0
Squamatic acid	57.05	150.59	390.34	2.33	4	9	6	0
Methylorsellinate	85.97	66.76	182.17	1.23	2	4	2	0
Zileuton *	76.30	94.80	236.29	1.23	2	4	2	0

Note: ^a^ Percentage of absorption (%ABS); ^b^ topological polar surface area (TPSA); ^c^ molecular weight (MW); ^d^ logarithm of partition coefficient between n-octanol and water (cLogP); ^e^ number of hydrogen bond donors (HBD); ^f^ number of hydrogen bond acceptors (HBA); ^g^ number of rotable bonds (n-ROTB). * Commercial redox inhibitors of 5-LOX.

**Table 5:** Structural fragments responsible for the toxicity of the compounds present in the species of the genus *Cladonia*.

Compound	Fraction of the Molecule	Risk of Toxicity
Usnic acid	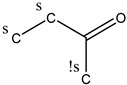	High-risk fragment indicating a reproductive effect
Squaric acid	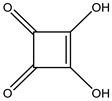	High-risk fragment indicating tumorigenicity
FP acid	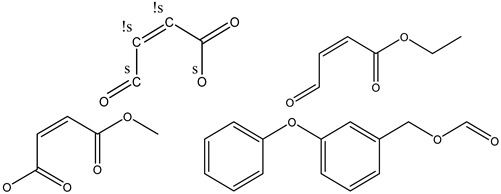	High-risk fragment indicating an irritant effect

Note: FP acid (furmarprotocetraric acid).

**Table 6:** Binding energies resulting from molecular docking experiments of the selected compounds in the extracts of *Cladonia*, together with the standard inhibitor zileuton on 5-lipoxygenase (5-LOX).

Compound	Binding Energy (Kcal/mol) 5-Lipoxygenase (5-LOX)
Thamnolic acid	−7.40
13D acid	−5.60
Perlatolic acid	−7.10
Psoromic acid	−8.30
Orsellinic acid	−5.60
Atranorin	−7.20
Cetraric acid	−7.60
Squamatic acid	−7.60
Methylorsellinate	−5.40
Zileuton	−8.70

## Data Availability

The data presented in this study are available on request from the corresponding authors.

## Sample Availability

Samples of the compounds are not available from the authors.

## References

[1] Grimm M., Grube M., Schiefelbein U., Zuhlke D., Bernhardt J., Riedel K. (2021). The lichens microbiota, still a mystery?. Front. Microbiol..

[2] Leiva D., Fernández-Mendoza F., Acevedo J., Carú M., Grube M., Orlando J. (2021). The Bacterial community of the foliose macro-lichen *Peltigera frigida* is more than a mere extension of the microbiota of the subjacent substrate. Microb. Ecol..

[3] Sancho L.G., Pintado A., Navarro F., Ramos M., De Pablo M.A., Blanquer J.M., Raggio J., Valladares F., Green T.G.A. (2017). Recent warming and cooling in the Antarctic Peninsula region has rapid and large effects on lichen vegetation. Sci. Rep..

[4] Johnson C.J., Bennett J.P., Biro S.M., Duque-Velasquez J.C., Rodriguez C.M., Bessen R.A., Rocke T. (2011). Degradation of the disease-associated prion protein by a serine proteasa from lichens. PLoS ONE.

[5] Redón J. (1985). Antarctic Lichens.

[6] Torres-Benítez A., Rivera-Montalvo M., Sepúlveda B., Castro O.N., Nagles E., Simirgiotis M.J., Garciá-Beltrán O., Areche C. (2017). Metabolomic analysis of two *Parmotrema* lichens: *P. robustum* (Degel.) Hale and *P. andinum* (Mull. rg.) hale using UHPLC-ESI-OTMS-MS. Molecules.

[7] Paudel B., Datta Bhattarai H., Prasad Pandey D., Seoun Hur J., Gyu Hong S., Kim I.C., Han Yim J. (2012). Antioxidant, antibacterial activity and brine shrimp toxicity test of some Mountainous lichens from Nepal. Biol. Res..

[8] Areche C., Parra J.R., Sepulveda B., García-Beltrán O., Simirgiotis M.J. (2022). UHPLC-MS metabolomic fingerprinting, antioxidant, and enzyme inhibition activities of *Himantormia lugubris* from Antarctica. Metabolites.

[9] Sinha S., Doble M., Manju S. (2019). 5-Lipoxygenase as a drug target: A review on trends in inhibitors structural design, SAR and mechanism based approach. Bioorg. Med. Chem..

[10] Gilbert N.C., Gerstmeier J., Schexnaydre E.E., Börner F., Garscha U., Neau D.B., Werz O., Newcomer M.E. (2020). Structural and mechanistic insights into 5-lipoxygenase inhibition by natural products. Nat. Chem. Biol..

[11] Lee S., Suh Y.J., Yang S., Hong D.G., Ishigami A., Kim H., Hur J.-S., Chang S.-C., Lee J. (2021). Neuroprotective and anti-inflammatory effects of evernic acid in an MPTP-induced Parkinson’s disease model. Int. J. Mol. Sci..

[12] Ureña-Vacas I., González-Burgos E., Divakar P.K., Gómez-Serranillos M.P. (2022). Lichen extracts from Cetrarioid clade provide neuroprotection against hydrogen peroxide-induced oxidative stress. Molecules.

[13] Sánchez-Rangel J.C., Benavides J., Heredia J.B., Cisneros-Zevallos L., Jacobo-Velázquez D.A. (2013). The Folin–Ciocalteu assay revisited: Improvement of its specificity for total phenolic content determination. Anal. Methods.

[14] Gómez J., Simirgiotis M., Lima B., Gamarra-Luques C., Bórquez J., Caballero D., Feresin G.E., Tapia A. (2019). UHPLC–Q/Orbitrap/MS/MS fingerprinting, free radical scavenging, and antimicrobial activity of *Tessaria absinthioides* (Hook. &Arn.) DC. (Asteraceae) lyophilized decoction from Argentina and Chile. Antioxidants.

[15] Karadag A., Ozcelik B., Saner S. (2009). Review of methods to determine antioxidant capacities. Food Anal. Methods.

[16] Parra C., Soto E., León G., Salas C.O., Heinrich M., Echiburú-Chau C. (2017). Nutritional composition, antioxidant activity and isolation of scopoletin from *Senecio nutans*: Support of ancestral and new uses. Nat. Prod. Res..

[17] Huang D., Ou B., Hampsch-Woodill M., Flanagan J.A., Prior R.L. (2002). High-throughput assay of oxygen radical absorbance capacity (ORAC) using a multichannel liquid handling system coupled with a microplate fluorescence reader in 96-well format. J. Agric. Food Chem..

[18] Brand-Willians W., Cuvelier M.E., Berset C.L.W.T. (1995). Use of a free radical method to evaluate antioxidant activity. LWT-Food Sci. Technol..

[19] Ellman G.L., Courtney K.D., Andres V., Featherstone R.M. (1961). A new and rapid colorimetric determination of acetylcholinesterase activity. Biochem. Pharmacol..

[20] Costamagna M.S., Zampini I.C., Alberto M.R., Cuello S., Torres S., Perez J., Quispe C., Schmeda-Hirschmann G., Isla M.I. (2016). Polyphenol rich fraction from *Geoffroea decorticans* fruits flour affects key enzymes involved in metabolic syndrome, oxidative stress and inflammatory process. Food Chem..

[21] Burgos-Edwards A., Jiménez-Aspee F., Thomas-Valdés S., Schmeda-Hirschmann G., Theoduloz C. (2017). Qualitative and quantitative changes in polyphenol composition and bioactivity of *Ribes magellanicum* and *R. punctatum* after in vitro gastrointestinal digestion. Food Chem..

[22] McDougall G.J., Kulkarni N.N., Stewart D. (2009). Berry polyphenols inhibit pancreatic lipase activity in vitro. Food Chem..

[23] Picot M.C.N., Mahomoodally M.F. (2017). Effects of *Aphloia theiformis* on key enzymes related to diabetes mellitus. Pharm. Biol..

[24] Azam F., Amer A., Abulifa A., Elzwawi M. (2014). Ginger components as new leads for the design and development of novel multi-targeted anti-Alzheimer’s drugs: A computational investigation. Drug Des. Dev. Ther..

[25] Ley-Martínez J.S., Ortega-Valencia J.E., García-Barradas O., Jiménez-Fernández M., Uribe-Lam E., Vencedor-Meraz C.I., Oliva-Ramírez J. (2022). Active compounds in *Zingiber officinale* as possible redox inhibitors of 5-lipoxygenase using an in silico approach. Int. J. Mol. Sci..

[26] Torres-Benítez A., Ortega-Valencia J.E., Sanchez M., Divakar P.K., Simirgiotis M.J., Gómez-Serranillos M.P. (2022). Metabolomic profiling, antioxidant and enzyme inhibition properties and molecular docking analysis of Antarctic lichens. Molecules.

[27] Chiasson A.I., Robichaud S., Moutombi F.J.N., Hébert M.P.A., Mbarik M., Surette M.E., Touaibia M. (2020). New zileutonhydroxycinnamic acid hybrids: Synthesis and structure-activity relationship towards 5-lipoxygenase inhibition. Molecules.

[28] Volkamer A., Kuhn D., Grombacher T., Rippmann F., Rarey M. (2012). Combining global and local measures for structure-based druggability predictions. J. Chem. Inf. Model..

[29] Muthuraman S., Sinha S., Vasavi C., Waidha K.M., Basu B., Munussami P., Balamurali M., Doble M., Kumar R.S. (2019). Design, synthesis and identification of novel coumaperine derivatives for inhibition of human 5-LOX: Antioxidant, pseudoperoxidase and docking studies. Bioorg. Med. Chem..

[30] Mosmann T. (1983). Rapid colorimetric assay for cellular growth and survival: Application to proliferation and cytotoxicity assays. J. Immunol. Methods.

[31] LeBel C.P., Ischiropoulos H., Bondy S.C. (1992). Evaluation of the probe 20,70-dichlorofluorescin as an indicator of reactive oxygen species formation and oxidative stress. Chem. Res. Toxicol..

[32] Sepúlveda B., Cornejo A., Bárcenas-Pérez D., Cheel J., Areche C. (2022). Two new fumarprotocetraric acid lactones identified and characterized by UHPLC-PDA/ESI/ORBITRAP/MS/MS from the Antarctic lichen *Cladonia metacorallifera*. Separations.

[33] Salgado F., Albornoz L., Cortéz C., Stashenko E., Urrea-Vallejo K., Nagles E., Galicia-Virviescas C., Cornejo A., Ardiles A., Simirgiotis M. (2018). Secondary metabolite profiling of species of the genus *Usnea* by UHPLC-ESI-OT-MS-MS. Molecules.

[34] Paukov A., Teptina A., Ermoshin A., Kruglova E., Shabardina L. (2022). The role of secondary metabolites and bark chemistry in shaping diversity and abundance of epiphytic lichens. Front. For. Glob. Chang..

[35] Phi K.-H., Shin M.-J., Lee S., So J.E., Kim J.H., Suh S.-S., Koo M.H., Shin S.C., Kim J.-H., Lee J.H. (2022). Bioactive terphenyls isolated from the Antarctic lichen *Stereocaulon alpinum*. Molecules.

[36] Aslan A., Güllüce M., Sökmen M., Adgüzel A., Sahin F., Özkan H. (2006). Antioxidant and antimicrobial properties of the lichens *Cladonia foliacea*, *Dermatocarpon miniatum*, *Everinia divaricata*, *Evernia prunastri*, and *Neofuscella pulla*. Pharm. Biol..

[37] Luo H., Yamamoto Y., Kim J., Jung J., Koh Y., Hur J. (2009). Lecanoric acid, a secondary lichen substance with antioxidant properties from *Umbilicaria antarctica* in maritime Antarctica (King George Island). Polar Biol..

[38] Kosanić M., Ranković B., Stanojković T., Rančić A., Manojlović N. (2014). *Cladonia* lichens and their major metabolites as possible natural antioxidant, antimicrobial and anticancer agents. LWT-Food Sci. Technol..

[39] Ranković B., Kosanić M., Stanojković T.P. (2011). Antioxidant, antimicrobial and anticancer activity of the lichens *Cladonia furcata*, *Lecanora atra* and *Lecanora muralis*. BMC Complement. Altern. Med..

[40] Singh S., Singh P., Ravindra R. (2011). Screening of antioxidant potential of Artic lichen. Polar Biol..

[41] Da Costa J., Rodrigues R., dos Santos C., Antoniolli A., de Souza A., Thomazzi S. (2010). Pharmacological properties of lichen *Cladonia clathrata*. Pharm. Biol..

[42] Gunasekaran S., Rajan V.P., Ramanathan S., Murugaiyah V., Samsudin M.W., Din L. (2016). Antibacterial and antioxidant activity of lichens *Usnea rubrotincta*, *Ramalina dumeticola*, *Cladonia verticillata* and their chemical constituents. Malays. J. Anal. Sci..

[43] Yücel O., Odabaşoǧlu F., Güllüce M., Çalik Z.Z., Çakir A., Aslan A., Yazici K., Halici M. (2007). Antioxidant and antimicrobial properties of a lichen species, *Cladonia rangiformis* growing in Turkey. Turk. J. Pharm. Sci..

[44] Prokop’ev I.A., Yatsyna A.P., Poryadina L.N., Filippova G.V., Shavarda A.L. (2018). Phenolic metabolites of lichens in the genus *Cladonia* growing in Belarus and Yakutia. Chem. Nat. Compd..

[45] Prokop’ev I.A., Filippova G.V. (2019). Antioxidant activity of secondary metabolites from *Cladonia* lichens. Chem. Nat. Compd..

[46] Paudel B., Bhattarai H.D., Lee J.S., Hong S.G., Shim H.W., Yim J.H. (2008). Antioxidant activity of polar lichens from King George Island (Antarctica). Polar Biol..

[47] Luo H., Li C., Kim J.C., Liu Y., Jung J.S., Koh Y.J., Hur J.S. (2013). Biruloquinone, an acetylcholinesterase inhibitor produced by lichen-forming fungus *Cladonia macilenta*. J. Microbiol. Biotechnol..

[48] Studzińska-Sroka E., Majchrzak-Celińska A., Zalewski P., Szwajgier D., Baranowska-Wójcik E., Kaproń B., Plech T., Żarowski M., Cielecka-Piontek J. (2021). Lichen-derived compounds and extracts as biologically active substances with anticancer and neuroprotective properties. Pharmaceuticals.

[49] Mukemre M., Zengin G., Turker R.S., Aslan A., Dalar A. (2021). Biological activities and chemical composition of *Xanthoria* lichens from Turkey. Int. J. Second. Metab..

[50] Devi A.P., Duong T.H., Ferron S., Beniddir M., Dinh M.H., Nguyen V.K., Pham N.K.T., Mac D.H., Boustie J., Chavasiri W. (2020). Salazinic acid-derived depsidones and diphenylethers with α-glucosidase inhibitory activity from the lichen *Parmotrema dilatatum*. Planta Med..

[51] Duong T.H., Hang T.X.H., Le Pogam P., Tran T.N., Mac D.H., Dinh M.H., Sichaem J. (2020). α-glucosidase inhibitory depsidones from the lichen *Parmotrema tsavoense*. Planta Med..

[52] White P.A.S., Oliveira R.C.M., Oliveira A.P., Serafini M.R., Araújo A.A.S., Gelain D.P., Moreira J.C.F., Almeida J.R.G.S., Quintans J.S.S., Quintans-Junior L.J. (2014). Antioxidant activity and mechanisms of action of natural compounds isolated from lichens: A systematic review. Molecules.

[53] Bézivin C., Tomasi S., Rouaud I., Delcros J.G., Boustie J. (2004). Citotoxic activity of compounds from the lichen: *Cladonia convolute*. Planta Med..

[54] Brisdelli F., Perilli M., Sellitri D., Piovano M., Garbarino J.A., Nicoletti M., Bozzi A., Amicosante G., Celenza G. (2012). Citotoxic activity and antioxidant capacity of purified lichen metabolites: An in vitro study. Phytother. Res..

